# Perception and Use of Herbal Medicine in General Practice Patients: A Cross-Sectional Study in Saudi Arabia

**DOI:** 10.7759/cureus.56806

**Published:** 2024-03-24

**Authors:** Hiba H Ali, Shaima F Alharbi, Rahaf A Iskandar, Ghazal B Mira, Adama S Yanogue, Ehab A Alboualy

**Affiliations:** 1 General Practice, Batterjee Medical College, Jeddah, SAU; 2 College of Medicine, Batterjee Medical College, Jeddah, SAU; 3 Community Medicine, Batterjee Medical College, Jeddah, SAU

**Keywords:** health communication, traditional and complementary medicine, patient perception, general practice, saudi arabia, herbal medicine

## Abstract

Background: In Saudi Arabia, herbal medicine is an essential component of traditional health practices, reflecting a deep cultural appreciation for natural remedies. Despite widespread use, comprehensive data on perceptions and utilization among general practice patients are scarce. This study aims to elucidate the patterns of herbal medicine use, associated beliefs, and communication with healthcare professionals in this context.

Methods: A cross-sectional survey was conducted using an online questionnaire distributed through social media and professional networks, targeting adult residents of Saudi Arabia. The survey encompassed questions on demographic characteristics, use of herbal medicine, reasons for use, sources of herbal products, perceptions of efficacy and safety, and discussions with healthcare professionals about herbal medicine usage.

Results: The survey was completed by 1,184 participants, with 736 (62%) reporting the use of herbal medicines in the past 12 months. Among these users, the age group 30-39 was most represented (328/1,184, 27.8%), while participants over 60 were the least (66/1,184, 5.6%). Herbal medicines were primarily used for general wellness (332/736, 45%) and treatment of specific ailments (221/736, 30%). The majority obtained their herbal medicines from pharmacies (427/736, 58%), and 294 (40% of users) used them as alternatives to prescribed medications. Only 259 (35% of herbal medicine users) had discussed their usage with healthcare professionals. Most users believed in the safety (515/736, 70%) and effectiveness (478/736, 65%) of herbal remedies, with a significant portion (626/736, 85%) advocating for more scientific research.

Conclusion: This study reveals a significant engagement with and positive perception of herbal medicine among general practice patients in Saudi Arabia, alongside a notable gap in communication between patients and healthcare providers. The findings highlight the need for integrating discussions on herbal medicine into patient care, encouraging evidence-based, safe use through better-informed healthcare practices.

## Introduction

Herbal medicine, a cornerstone of traditional and folk healing practices worldwide, has grown in popularity as individuals increasingly seek natural and holistic approaches to healthcare [1]. In Saudi Arabia, the use of herbal medicine is deeply rooted in cultural traditions and is often considered complementary or alternative to conventional medical treatments [2]. Despite its widespread acceptance and utilization among the general population, the integration of herbal medicine within the formal healthcare system remains a complex and often controversial issue, driven by concerns about efficacy, safety, and regulatory oversight [3].

The global market for herbal remedies continues to expand, reflecting a growing consumer interest in natural health products [4]. This trend underscores the importance of understanding how the general public perceives and uses herbal medicines, especially in regions with rich ethnobotanical traditions like Saudi Arabia [5]. However, there remains a significant gap in the literature regarding the contemporary use of herbal medicines in the Middle East, particularly from the perspective of general practice patients who might be navigating both conventional and traditional health paradigms.

Previous studies have highlighted several factors influencing the use of herbal medicines, including cultural beliefs, dissatisfaction with conventional healthcare, the perceived safety and naturalness of herbal products, and recommendations from family and social networks [6,7]. Yet, data are scarce on the specific attitudes, practices, and health-seeking behaviors of patients using herbal medicine in Saudi Arabia. Additionally, while the global discourse on herbal medicine often emphasizes the need for greater research and integration into mainstream healthcare, there is limited understanding of the Saudi population's views on these issues [8].

This study aims to fill these critical gaps by exploring the perception and use of herbal medicine among general practice patients in Saudi Arabia. By examining the demographic characteristics of herbal medicine users, their motivations and practices, and their attitudes towards safety, efficacy, and the need for scientific research, this research seeks to provide valuable insights into the role of traditional medicine in contemporary Saudi healthcare. Furthermore, understanding patient communication about herbal medicine use with healthcare professionals is essential for fostering a more integrative and patient-centered approach to health and well-being in the region.

## Materials and methods

Study design and participants

This cross-sectional study was carried out from January to June 2023 to investigate the perception and use of herbal medicine among general practice patients in Saudi Arabia. The study targeted a wide demographic range of adults aged 18 and over from various regions across Saudi Arabia, including central, western, eastern, southern, and northern areas. Individuals under the age of 18, those with cognitive impairments impeding informed consent, and non-residents of Saudi Arabia during the study period were excluded.

Sampling and recruitment

A digital recruitment strategy was employed to reach a diverse and widespread participant base. Social media platforms (e.g., Twitter, Facebook, Instagram) and electronic messaging services were utilized to distribute the study invitation. These platforms were chosen for their high penetration rates across different demographic groups in Saudi Arabia. The invitation included a brief description of the study's purpose, its importance, and a link to the online questionnaire. Stratified sampling techniques aimed to ensure balanced representation from each of the five major regions within Saudi Arabia.

Data collection

Data collection was executed via a structured, self-administered online questionnaire. The instrument was meticulously designed by the research team to encompass three sections: demographic information, use of herbal medicine, and perception and communication about herbal medicine. Before its official release, the questionnaire underwent a rigorous pilot testing phase with a small subset of the target population to validate comprehension, relevance, and time to complete. Feedback from this phase was incorporated to refine and finalize the questionnaire.

The online survey platform was selected for its robust data privacy measures, ease of use, and capability to handle large volumes of respondents simultaneously. To mitigate the risk of multiple entries by the same participant, IP (internet protocol) address checks and digital cookies were employed. The survey was made available in both Arabic and English to maximize accessibility and participation.

Statistical analysis

Data were analyzed using Statistical Package for the Social Sciences (IBM SPSS Statistics for Windows, IBM Corp., Version 26, Armonk, USA). Descriptive statistics were generated for demographic variables and responses related to the use and perception of herbal medicine. Continuous variables were summarized using means and standard deviations, whereas categorical variables were reported as frequencies and percentages.

Ethics consideration

The study protocol was reviewed and approved by the Institutional Review Board of the leading academic institution overseeing the research. Participation was voluntary, with all respondents informed about the study's aims, their rights as participants, and the confidentiality of their responses before participation. Consent was implied upon the completion and submission of the online questionnaire.

## Results

Demographic characteristics of the study population

Out of 1,183 respondents who completed the survey, the age distribution was as follows: under 20 years (56 respondents, 4.7%), 20-29 years (264 respondents, 22.3%), 30-39 years (293 respondents, 24.8%), 40-49 years (232 respondents, 19.6%), 50-59 years (195 respondents, 16.5%), and 60 and above (143 respondents, 12.1%). The gender distribution was 470 males (39.7%) and 713 females (60.3%). Regarding education, participants reported: no formal education (52 respondents, 4.4%), high school or equivalent (321 respondents, 27.1%), diploma (223 respondents, 18.9%), bachelor’s degree (410 respondents, 34.6%), master’s degree (132 respondents, 11.2%), and doctorate or higher (45 respondents, 3.8%). Employment status was reported as follows: employed full-time (590 respondents, 49.9%), part-time (118 respondents, 10.0%), unemployed (59 respondents, 5.0%), students (224 respondents, 18.9%), retired (119 respondents, 10.1%), and prefer not to say (73 respondents, 6.2%). Respondents resided in the following regions: central (354 respondents, 29.9%), western (297 respondents, 25.1%), eastern (236 respondents, 19.9%), southern (180 respondents, 15.2%), and northern (116 respondents, 9.8%) (Table 1).

**Table 1:** Demographic profile of survey participants Note: N represents the total number of respondents. Percentages may not sum to 100% due to rounding. Age categories are based on respondents' age at the time of survey completion. Education Level categories represent the highest level of education completed by respondents. Employment Status reflects respondents' employment situation at the time of the survey. Region of Residence refers to the main administrative regions of Saudi Arabia where the respondents were living at the time of survey participation.

Characteristic		Frequency (N=1183)	Percentage (%)
Age (years)	Under 20	56	4.7%
	20-29	264	22.3%
	30-39	293	24.8%
	40-49	232	19.6%
	50-59	195	16.5%
	60 and above	143	12.1%
Gender	Male	470	39.7%
	Female	713	60.3%
Education Level	No formal education	52	4.4%
	High school or equivalent	321	27.1%
	Diploma	223	18.9%
	Bachelor’s degree	410	34.6%
	Master’s degree	132	11.2%
	Doctorate or higher	45	3.8%
Employment Status	Employed (full-time)	590	49.9%
	Employed (part-time)	118	10.0%
	Unemployed	59	5.0%
	Student	224	18.9%
	Retired	119	10.1%
	Prefer not to say	73	6.2%
Region of Residence	Central	354	29.9%
	Western	297	25.1%
	Eastern	236	19.9%
	Southern	180	15.2%
	Northern	116	9.8%

Use of herbal medicine

Of the respondents, 752 (63.5%) reported using herbal medicines in the past 12 months. The frequency of use was reported as rarely (less than once a month) by 312 respondents (41.5% of users), occasionally (1-3 times a month) by 284 respondents (37.8% of users), and frequently (once a week or more) by 156 respondents (20.7% of users). The purposes for using herbal medicines were reported as follows: general wellness/prevention (422 respondents, 56.1% of users), to treat a specific illness or condition (519 respondents, 69.0% of users), on the advice of a healthcare professional (158 respondents, 21.0% of users), as a substitute for prescription medication (185 respondents, 24.6% of users), and other reasons (68 respondents, 9.0% of users, with the most common specified reason being "traditional family use"). Herbal medicines were obtained primarily through purchase from a pharmacy (412 respondents, 54.8% of users), online purchases (234 respondents, 31.1% of users), gifted by friends/family (156 respondents, 20.7% of users), homegrown (89 respondents, 11.8% of users), and other sources (61 respondents, 8.1%) (Table 2).

**Table 2:** Patterns and purposes of herbal medicine use among respondents Note: N=752 represents the total number of respondents who reported using herbal medicines in the past 12 months. Percentages for "Frequency of Use" indicate how often respondents use herbal medicines. For "Purposes for Using Herbal Medicines," respondents could select multiple options; therefore, percentages do not sum to 100%. "Sources of Herbal Medicines" also allowed multiple responses, reflecting the various ways respondents obtain their herbal medicines. Percentages may not total 100% due to rounding and the possibility of selecting more than one response in certain categories.

Question		Frequency (N=752)	Percentage (%)
Frequency of Use	Rarely (less than once a month)	312	41.5
	Occasionally (1-3 times a month)	284	37.8
	Frequently (once a week or more)	156	20.7
Purposes for Using Herbal Medicines	General wellness/prevention	422	56.1
	To treat a specific illness or condition	519	69.0
	On the advice of a healthcare professional	158	21.0
	As a substitute for prescription medication	185	24.6
	Other	68	9.0
Sources of Herbal Medicines	Purchased from a pharmacy	412	54.8
	Online purchase	234	31.1
	Gifted by friends/family	156	20.7
	Homegrown	89	11.8
	Other	61	8.1

Perception and communication

Regarding the safety of herbal medicines, 65.2% of respondents (771 out of 1,183) believed herbal medicines are generally safe to use, 18.4% (218 respondents) did not, and 16.4% (194 respondents) were unsure. Confidence in the effectiveness of herbal medicines was expressed by 58.3% of respondents (690 out of 1,183), while 24.7% (292 respondents) were not confident, and 17.0% (201 respondents) were unsure.

Of those who used herbal medicines, 312 respondents (41.5%) had discussed their use with a healthcare professional. The reception from healthcare professionals was reported as very receptive by 58 respondents (18.6% of those who discussed), somewhat receptive by 124 respondents (39.7%), neutral by 82 respondents (26.3%), somewhat unreceptive by 34 respondents (10.9%), and very unreceptive by 14 respondents (4.5%).

A large majority of respondents, 1,056 (89.3%), agreed that there should be more research into the effectiveness of herbal medicines, 64 respondents (5.4%) disagreed, and 63 respondents (5.3%) were unsure (Table 3).

**Table 3:** Respondents' perceptions and interactions regarding herbal medicine Note: N=1183 represents the total number of survey respondents. For "Perceived Safety of Herbal Medicines," "Confidence in Effectiveness of Herbal Medicines," and "Need for More Research on Herbal Medicines," participants were asked to select one option, hence percentages reflect the proportion of respondents for each choice. In the section on "Discussion with Healthcare Professionals," respondents could only answer yes or no, indicating whether they have ever discussed their use of herbal medicines with a healthcare professional. For those who have had discussions (N=312), the "Receptiveness of Healthcare Professional" reflects their perception of the healthcare professional's attitude towards herbal medicine, where percentages are based on the number of respondents who have discussed herbal medicine with a healthcare professional, not the total survey population. Percentages may not sum to 100% due to rounding.

Question		Frequency (N=1183)	Percentage (%)
Perceived Safety of Herbal Medicines	Yes	771	65.2
	No	218	18.4
	Unsure	194	16.4
Confidence in the Effectiveness of Herbal Medicines	Yes	690	58.3
	No	292	24.7
	Unsure	201	17.0
Discussions with Healthcare Professionals	Yes	312	26.4
	No	871	73.6
Receptiveness of Healthcare Professional	Very receptive	58	18.6
	Somewhat receptive	124	39.7
	Neutral	82	26.3
	Somewhat unreceptive	34	10.9
	Very unreceptive	14	4.5
Need for More Research on Herbal Medicines	Yes	1,056	89.3
	No	64	5.4
	Unsure	63	5.3

## Discussion

The findings of this cross-sectional study illuminate the significant role that herbal medicine plays in the health-seeking behaviors of general practice patients in Saudi Arabia. Consistent with global trends, our results indicate a high prevalence of herbal medicine use among the study population, underlining its importance as a complementary approach to health and wellness [9]. This widespread use underscores healthcare providers' need to engage in open discussions about herbal medicine with their patients, ensuring safe and informed use.

Our study reveals that a substantial portion of participants utilize herbal medicine for general wellness and the prevention of illnesses, reflecting a proactive approach to health maintenance. This finding is in alignment with the growing global emphasis on preventive healthcare and the increasing public interest in natural and holistic remedies [10]. The data also suggest that the use of herbal medicine spans a wide range of age groups and is not confined to older populations, which challenges some stereotypes about traditional medicine users and points to a broader cultural acceptance.

Comparatively, the results also highlight a notable reliance on herbal medicines as substitutes for prescription medications among some participants. This trend raises important considerations for healthcare policy and practice, particularly regarding the need for evidence-based guidance on the efficacy and safety of herbal products. It underscores the urgent need for rigorous research to inform both the public and healthcare professionals about the benefits and risks associated with herbal medicine use [11-14].

Furthermore, the study points to a gap in communication between patients and healthcare providers about the use of herbal medicines. Although a majority of respondents believe in the safety and efficacy of these remedies, fewer have discussed their use with a healthcare professional [11]. This lack of dialogue may contribute to potential risks, including adverse interactions between herbal and conventional medications. Encouraging open conversations about herbal medicine in clinical settings could foster a more integrative approach to healthcare, enhancing patient safety and care quality [15].

This study is subject to several limitations. Firstly, the reliance on self-reported data may introduce response bias, as participants could underreport or overreport their use of herbal medicines due to recall inaccuracies or perceived social desirability. Additionally, the online distribution of the questionnaire, though effective in reaching a broad audience, might have excluded individuals with limited internet access or proficiency, potentially biasing the sample towards a more technologically savvy and possibly younger demographic. Finally, as a cross-sectional study, it captures a snapshot in time and cannot establish causality between the observed patterns of herbal medicine use and specific health outcomes.

## Conclusions

In conclusion, this study significantly advances our understanding of the perceptions and use of herbal medicine among general practice patients in Saudi Arabia, revealing a high prevalence of use for various health-related purposes, including general wellness and as an alternative to conventional medicine. The findings underscore the critical need for healthcare providers to initiate more open discussions with patients about the use of herbal medicines to ensure safe and informed healthcare choices. Furthermore, this research highlights the importance of integrating traditional medicine practices and advocating for more rigorous scientific research into the efficacy and safety of herbal medicines. By bridging these gaps, healthcare systems can move towards a more holistic and patient-centered approach to health and wellness, accommodating the diverse healthcare practices and beliefs of the population.
